# Supplementary material for: MetaRibo-Seq measures translation in microbiomes
Source: Nat Commun. 2020 Jun 29;11:3268. doi: 10.1038/s41467-020-17081-z (PMC7324362; doi:10.1038/s41467-020-17081-z)
Supplement: Supplementary file 10 — Supplementary Data 7 [file 41467_2020_17081_MOESM10_ESM.zip › File2/Confidence_High_Taxonomy/109077_out.krona.html]

Javascript must be enabled to view this page.

members
magnitude
magnitudeUnassigned
count
unassigned
taxon
rank

109077\_out

3

superkingdom
3
2

3
1239
phylum

3
186801
class

order
3
186802

family
541000
3

species

SRS013098\_contig\_number\_17006SRS052697\_contig\_number\_contig-100\_4183.185892SRS142503\_contig\_number\_30248
3
1898205
